# Evaluation of a Dental Diagnostic Terminology Subset

**DOI:** 10.3233/SHTI190555

**Published:** 2019-08-21

**Authors:** Heather L. Taylor, Zasim Siddiqui, Kendall Frazier, Thankam Thyvalikakath

**Affiliations:** a Richard M. Fairbanks School of Public Health, IUPUI, Indianapolis, Indiana, USA; b Indiana University School of Dentistry, Indianapolis, Indiana, USA; c Center for Biomedical Informatics, Regenstrief Institute, Indianapolis, Indiana, USA

**Keywords:** Electronic Health Record, Electronic Dental Records, SNOMED-CT

## Abstract

The objective of this study was to determine how well a subset of SNODENT, specifically designed for general dentistry, meets the needs of dental practitioners. Participants were asked to locate their written diagnosis for tooth conditions among the SNODENT terminology uploaded into an electronic dental record. Investigators found that 65% of providers’ original written diagnoses were in “agreement” with their selected SNODENT dental diagnostic subset concept(s).

## Introduction

Recently, SNODENT (a subset of SNOMED CT since 2013) and DDS (an interface dental terminology) were harmonized [1–3]. The combination of these two terminologies led to the 2017 accreditation of *SNODENT, a dental diagnostic terminology* as the standard by the American National Standard Institute (ANSI) and the American Dental Association (ADA), and the creation of two subsets, SNO-DDS and SNO-DDS General Dentistry [2,3]. To date, *SNODENT, a dental diagnostic terminology*, or any of its subsets, have yet to be thoroughly evaluated for content coverage and completeness. The ADA Practice Institute developed a subset through expert opinion and consensus for use in the dental clinics of the School of Dentistry, University of Detroit Mercy, MI. The subset was developed to facilitate efficient documentation of common dental conditions seen in a general dentistry setting. Therefore, the objective of this study was to determine how well this subset of SNODENT met the needs of dental practitioners.

## Methods

A subset of the SNODENT terminology (410 unique concepts), was uploaded into the training module of axiUm 6.x (Exan corporation, Vancouver, BC, Canada), the Indiana University School of Dentistry’s electronic dental record (EDR).

We recruited a convenience sample of 20 participants, consisting of six faculty and fourteen third-year and fourth-year dental students. Participants were either full-time clinical faculty or third-year or fourth-year dental students to ensure they were familiar with documenting patient care in the EDR system.

Investigators selected a record describing a dental case-patient (herein referred to as “case-patient”), originally explained in a previous study [4]. The record included the case patient’s health history information and oral findings related to periodontal disease and caries. Specifically, the case-patient had poor oral hygiene, generalized gingival inflammation, mesial and distal primary caries on anterior teeth, cracked teeth, secondary caries, extensive decay with pulp exposure, and a periapical radiolucency.

Upon enrollment into the study, participants were asked to review the record and to “think aloud” as they examined the case patient’s medical history, dental findings, clinical photographs, and radiographs. The “think aloud” method includes the participant verbalizing their thoughts and actions as they carry out tasks [5–7] The entire session was audio-and video-recorded to capture participants’ interactions and thoughts. Each participant thought aloud while reviewing the case-patient record and while writing dental diagnoses for eight teeth and the overall gingival health of the case-patient on a paper form.

Continuing to “think aloud,” participants next worked within the treatment planning module of the EDR to locate and select the “best” SNODENT diagnostic terminology(s) for each of their written diagnoses. Afterward, participants rated their satisfaction on their selected SNODENT concept and its ability to represent their original written diagnosis. Specifically, each participant was asked to determine if they were “completely,” “partially,” or “not at all” satisfied with each SNODENT concept selected within the EDR.

In addition to participant observation, we recorded participants’ interactions with the EDR using screen and voice capturing software Camtasia® (TechSmith Corporation, Okemos, MI, USA). At the end of the study session, each participant completed a questionnaire consisting of four Likert scale questions as well as two open-ended questions. The questionnaire was designed to assess the participants’ opinions on the use and clinical value of the subset presented to them in the EDR.

### Data Analysis

Two investigators (HT & ZS) independently compared each participants’ written diagnoses and the corresponding SNODENT concept selected from the subset of SNODENT concepts uploaded into the EDR. Written diagnoses and selected SNODENT concepts were considered in “agreement” if they represented analogous clinical meanings. Conversely, if the written diagnosis and the selected SNODENT concept were different in meaning or intent, the match was labeled as “non-agreement” by investigators. Differences between the two investigators’ classification of “agreement” and “non-agreement” cases were resolved through discussion with a third investigator (TT). Inter-rater reliability between the two investigators was calculated using Cohen’s Kappa coefficient. Further, the percentage of SNODENT concept selections deemed in “agreement” with the participant’s written diagnoses was calculated. In addition, the percentage of agreement among subgroups (students versus faculty) was compared using a generalized estimating equation (GEE) model for logistic regression.

We calculated the overall percentage of participant satisfaction with their selected SNODENT concept. Students’ satisfaction with their selections was compared to faculty’ satisfaction using a GEE model for ordinal logistic regression. Regressions were performed with a 95% confidence interval at a p-value of 0.05. The software IBM SPSS Statistics Version 23 (SPSS, Inc., Chicago, IL, USA) was used to perform statistical analysis. Descriptive statistics were utilized to evaluate the Likert scale questions of the end questionnaire. Qualitative content analysis was used to determine any recurring themes in the open-ended responses of the end questionnaire. The recordings were evaluated to determine what barriers, if any, limited the participant’s experience using the subset of SNODENT concepts within the EDR.

## Results

Twenty participants selected a total of 251 SNODENT concepts (42 unique codes) to diagnose the specified dental conditions of the case-patient. Study investigators compared participants’ written diagnosis and the selected SNODENT subset concept(s) for similarity in conceptual representation. Inter-rater reliability between the two investigators was 89%.

Investigators found that 162 (64.5%) of the written diagnostic concepts were in “agreement,” and 89 concepts (35.5%) were in “non-agreement” with the participants’ corresponding selected SNODENT concept(s).

The subgroup analysis revealed that students selected a total of 169 diagnostic concepts and had 75% “agreement” between their written diagnoses and their selected SNODENT subset concepts, whereas faculty selected a total of 82 diagnostic concepts and had 44% “agreement.” The percentage of concept “agreement” among students was significantly higher than faculty (p=0.0270, odds ratio 3.1).

Participants’ were asked to subjectively report their satisfaction with the SNODENT concept(s) and its representation of their original diagnosis. They reported “completely” satisfied with 155 (62%) of their selected SNODENT subset concepts, “partially” satisfied with 82 (32.5 %) and “not at all” satisfied with 14 (5.5%) of their selections. No significant differences were observed in satisfaction levels between students and faculty (p=0.54, odds ratio 1.5). Our end study questionnaire revealed that participants perceived value in this particular subset of the SNODENT terminology.

Analysis of the participants’ open-ended responses to the end questionnaire revealed issues with the subset of SNODENT concepts and the EDR interface. Participants who were frustrated with the terminology reported that there were too many concepts to search through, too many options with similar meanings, and concepts missing. Regarding the EDR interface, participants noted their frustration with the categorization of certain concepts, the necessity to search for the “exact” concept with correct spelling (the EDR search toolbox offered no suggestions for misspelled words), and the time required to locate concepts.

Video analyses revealed that 75% (15) of participants experienced difficulty finding all of their written diagnoses within the subset of SNODENT concepts. To compensate, participants found substitute diagnostic concepts for the majority of their original diagnoses. For 11 searches, participants were not able to locate a substitute. Both students and faculty had difficulty in determining which SNODENT concept within the subset was the “best” selection, especially among concepts with similar meaning.

## Conclusions

The majority of participants in this study agreed that a subset of SNODENT concepts within the EDR could add value to patient care and treatment planning. Investigators found that 65% of providers’ original written diagnoses were in “agreement” with their selected SNODENT dental diagnostic subset concept(s). Our findings illuminate the need for continual improvement of dental diagnostic terminologies through revisions and updates. We recommend training on the use of dental diagnostic terminologies for documentation of dental diagnoses, and findings for all dental providers. In addition, an intuitive user interface has a major role in supporting accurate and complete documentation of diagnosis and findings using controlled terminologies.
